# Comparison of general practitioners and rheumatologists' prescription patterns for patients with knee osteoarthritis

**DOI:** 10.1186/1471-2474-12-72

**Published:** 2011-04-12

**Authors:** Pascal Richette, Pascal Hilliquin, Philippe Bertin, Paolo Carni, Véronique Berger, Marc Marty

**Affiliations:** 1 Univ Paris Diderot, Sorbonne Paris Cité, Assistance Publique-Hôpitaux de Paris, Hôpital Lariboisière, Fédération de Rhumatologie, 75010, Paris, France; 2 Service de Rhumatologie, Centre hospitalier Sud Francilien, Corbeil-Essonnes, France; 3 Service de Rhumatologie, Hôpital Dupuytren, Limoges, France; 4 Service de Biométrie, Cenbiotech et Ceren, Dijon, France; 5 Nukleus, Département Etudes Cliniques, Paris, France; 6 Université Paris 12, UFR médicale, Assistance Publique-Hôpitaux de Paris, Service de Rhumatologie, Hôpital Henri Mondor, Créteil, France

## Abstract

**Background:**

To compare the prescription modalities of general practitioners (GPs) and rheumatologists (RHs) for symptomatic knee osteoarthritis (OA) and to determine correlates with prescription of low-dose NSAIDs.

**Methods:**

This observational, prospective, national survey was carried out among a national representative sample of GPs (n = 808) and RHs (n = 134). Each physician completed a medical questionnaire for the 2 most recent patients fulfilling the ACR criteria for knee OA.

**Results:**

GPs and RHs included 1,570 and 251 patients, respectively. Mean pain level of the knee (on a VAS, 0-100 mm) was greater for GP patients than for RH patients (49.8 ± 16.3 vs. 46.2 ± 17.1 mm, respectively; p < 0.01). As compared with patients of RHs, those of GPs more frequently had another joint affected by OA: 71.2% vs. 63.7% (p < 0.0001) and more often had hypertension and diabetes mellitus (p < 0.05).

As compared with RHs, GPs more frequently prescribed low-dose NSAIDs (p < 0.0001), oral NSAIDs (p < 0.05), and topical NSAIDs (p < 0.0001) but less frequently symptomatic slow-acting drugs for OA (p < 0.01). Moreover, GPs more frequently recommended rehabilitation (p < 0.01) and loss of weight (p < 0.0001). Logistic regression analysis revealed an association of low-dose NSAIDs prescription and prescription by GPs, prescription of topical NSAIDs, no prescription of oral NSAIDs or coxibs and no intra-articular injection of steroids.

**Conclusions:**

This study identified speciality-related variability in some aspects of the management of knee OA. The clinical profile of patients with knee OA differed between GPs and RHs.

## Background

Osteoarthritis (OA) is the most prevalent joint disease worldwide and a leading cause of chronic disability [1]. The prevalence of symptomatic knee OA has been recently estimated to be 9% in the general population. This estimate was computed with a Kellgren-Lawrence score of ≥1 used to define OA [2]. France has about 40-fold more general practitioners (GPs; n = 101,667 in 2009) than rheumatologists (RHs; n = 2,625 in 2007) and thus, most symptomatic knee OA is managed by GPs.

Several recommendations have been published for the treatment of knee OA, including non pharmacological and pharmacological treatments, for both GPs and RHs [3-5]. Use of these treatments may differ among GPs and RHs, but few studies [6] have investigated the prescription patterns of these two types of physicians for the treatment of knee OA.

Because the prevalence of knee OA increases with age, the safety of prescribed drugs deserves special consideration, and the benefits and risks of prescribing nonsteroidal anti-inflammatory drugs (NSAIDs) must be cautiously evaluated for each patient. Indeed, serious side effects occur with the long-term use of NSAIDs by elderly people with OA. The drugs can cause severe gastrointestinal complications, such as bleeding or perforation [7]. Several studies and meta-analyses have also suggested an association of increased cardiovascular risk and the use of traditional NSAIDs and coxibs [8,9]. Because of the dose-dependent elevation in cardiovascular and gastrointestinal risks [7,9], NSAIDs should be used at the lowest effective dose for the shortest possible time [4]. Use of NSAIDs at a low dose, such as ibuprofen at up to 1200 mg or naproxen at up to 500 mg, may be associated with decreased cardiovascular risk [9] and may be an interesting alternative to alleviate pain in patients with OA. Indeed ibuprofen (400 mg 3 times daily) has been shown to be more effective than acetaminophen (1000 mg 3 times daily) in reducing pain and improving function in patients with knee or hip OA [10].

We designed this national observational prospective study to compare the prescription patterns of GPs and RHs for patients with knee OA, with a special emphasis on low-dose NSAIDs prescriptions.

We found that there is speciality-related variability in some aspects of the management of knee OA and that the clinical profile of patients with knee OA differed between GPs and RHs.

## Methods

### Design

The study took the form of a cross-sectional survey by questionnaires completed by GPs and RHs working full- or part-time in different areas of France. This study was conducted in accordance with the recommendations of the Helsinki declaration. Ethical approval was not required for this study, in accordance with national guidelines.

### Selection of physicians

GPs were randomly selected from the CEGEDIM registry and RHs from the French Society of Rheumatology registry. In total, 7,451 GPs and 1,777 RHs in private practice were asked to participate; 1,194 GPs (16.0%) and 225 RHs (12.7%) agreed and were sent questionnaires in May 2008. Finally, 808 GPs (67.6%) and 134 RHs (59.5%) recruited patients. This rate of participation was expected and is usual for this kind of survey. The demographics of these GPs and RHs did not significantly differ from those of GPs and RHs in France in general in terms of sex, age and number of years of practice.

### Patients

Participating GPs and RHs were asked to record data for 2 consecutive patients presenting symptomatic knee OA according to the American College of Rheumatology criteria [11]. Eligible patients with knee OA were ≥ 18 years old and did not have another condition that might have interfered with the assessment of knee OA.

### Questionnaire

After a clinical examination, physicians were asked to complete a questionnaire covering the clinical characteristics of OA, co-morbidities, current medications and the different treatments prescribed for knee OA at the end of the consultation. In France, low-dose NSAIDs are sold over the counter and are ibuprofen (up to 1200 mg), ketoprofen (up to 75 mg/j) and naproxen (up to 660 mg/j). Additionally, available SYSADOA are avocado/soybean unsaponifiable, chondroitin, glucosamine and diacerein. The questionnaire was pragmatic and did not provide the definition of the recorded co-morbidities (hypertension, diabetes mellitus, peptic ulcer, cardiovascular risk, history of cancer, anxiety, depression) to avoid any bias in the prescription. Pain intensity was assessed by a 100-mm visual analog scale (VAS). The patients were also asked to report the presence or absence of daily disability due to knee OA (yes/no) and the presence or absence of pain at night (yes/no). A pilot study included a group of 2 GPs and 2 RHs, as well as a group of 4 clinical and non-clinical researchers experienced in this type of survey, to test the questionnaire for comprehensibility and relevance.

### Statistical analysis

Data are reported as mean ± standard deviation (SD) or percentages. Comparisons between the data for GPs and RHs involved the chi-square test or *t *test, as appropriate. Multiple regression analysis was used to assess the independent association of the prescription of low-dose NSAIDs and other variables. Because the study was exploratory, the sample size was calculated to provide a satisfying precision in the worst situation for ordinal data (rate of 50%). With an alpha risk of 5% and an expected precision of 2.5%, the number of patients needed was 1,536 and therefore 768 practitioners (who would each recruit 2 patients). Because the expected rate of practitioners actively recruiting patients among those giving their consent to participate was 55%, we invited 1,400 physicians (1,200 GPs and 200 RHs) to participate. A P value < 0.05 was considered statistically significant. All analyses involved use of SAS v9.2 (SAS Inst., Cary, NC).

## Results

### Demographic characteristics of GPs and RHs

The 808 GPs and 134 RHs not differ in age (52.2 ± 6.8 vs. 52.7 ± 7.5 years) (Table 1). However, GPs were more frequently male (89.4% vs. 67.2%; p < 0.0001), more often practiced in rural areas (55.0% vs. 19.4%; p < 0.0001) and saw fewer patients with knee OA per week than did RHs (11.1 ± 8.6 vs. 16.7 ± 8.5; p < 0.0001).

**Table 1:** Characteristics of general practitioners (GPs) and rheumatologists (RHs)

	GPs n = 808	RHs n = 134	P value
Age, years	52.2 ± 6.8	52.7 ± 7.5	ns
Sex, % male	89.4	67.2	< 0.0001
Practice location: % metropolitan/rural	45.0/55.0	80.6/19.4	< 0.0001
Number of patients seen per week	141.1 ± 39.3	91.2 ± 30.0	< 0.0001
Number of patients with knee OA seen per week	11.1 ± 8.6	16.7 ± 8.5	< 0.0001

Values are mean ± SD or percentage. ns = not significant.

### Demographic characteristics of patients with knee OA

GPs and RHs included 1,570 and 251 patients with knee OA, respectively (Table 2). As compared with patients of RHs, those of GPs had longer duration of OA (7.8 ± 5.6 vs. 6.8 ± 5.4 years; p < 0.01), more often experienced pain at rest and on movement (62.4 ± 17.6 vs. 57.3 ± 19.0 mm; p < 0.0001), and more frequently reported pain at night (49.9% vs. 29.7%; p < 0.0001) and daily disability due to knee OA (90.6% vs. 84.3%; p < 0.01). As compared with RH patients, GP patients more often had another joint (hand or hip) affected by OA: 71.2% vs. 63.7% (p < 0.0001). The presence of one or more pathological conditions was detected in 94.1% of patients. The most frequent severe comorbidities were hypertension (57.3%), diabetes mellitus (16.1%) and peptic ulcer (12.7%). The 2 groups of patients differed in relative frequencies of comorbid conditions (Table 3): hypertension and diabetes mellitus were more frequent in GP patients (p < 0.05 for both).

**Table 2:** Characteristics of patients with knee OA seen by general practitioners (GPs) and rheumatologists (RHs)

	All patients n = 1,821	GPs n = 1,570	RHs n = 251	P value
Age, years	67.3 ± 9.7	67.0 ± 9.7	69.8 ± 9.7	< 0.0001
Sex, % female	56.2	55.2	62.2	< 0.05
BMI, kg/m^2^	28.9 ± 4.7	29.1 ± 4.6	28.0 ± 4.9	< 0.01
Duration of disease, years	7.7 ± 5.7	7.8 ± 5.6	6.8 ± 5.4	< 0.01
Pain (VAS, 0-100 mm)				
On movement over the last 24 hr	61.6 ± 17.9	62.4 ± 17.6	57.3 ± 19.0	< 0.0001
At rest	32.7 ± 20.0	33.9 ± 20.5	25.3 ± 20.6	< 0.0001
Global pain	49.3 ± 16.4	49.8 ± 16.3	46.2 ± 17.1	< 0.01
Pain at night, %	47.1	49.9	29.7	< 0.0001
Daily disability, %	89.7	90.6	84.3	< 0.01
Presence of SF effusion, %	30.0	29.2	35.8	< 0.05

Values are mean ± SD or percentage. BMI = body mass index; VAS = visual analog scale; SF = synovial fluid.

**Table 3:** Co-morbidities in patients with knee OA seen by general practitioners (GPs) and rheumatologists (RHs)

	All patients n = 1,821	GPs n = 1,570	RHs n = 251	P value
Hypertension	57.3	58.3	51.0	< 0.05
Diabetes mellitus	16.1	16.9	11.6	< 0.05
Peptic ulcer	12.7	11.9	17.5	< 0.05
Cardiovascular risk	27.5	28.2	23.5	ns
History of cancer	5.2	5.2	5.2	ns
Anxiety	21.8	22.9	14.7	< 0.01
Depression	12.2	12.7	8.8	ns

Values are percentages. ns = not significant

### Prescription modalities of GPs and RHs

A high proportion of patients (80.8%) were receiving a mean of 3.6 ± 2.2 medications for another condition, and 4.5% were receiving oral anticoagulants. GPs and RHs prescribed drugs for 89.0% and 83.3% of their patients, respectively (p < 0.0001) (Table 4). GPs more frequently than RHs prescribed low-dose NSAIDs (15.1% vs. 5.2%; p < 0.0001), oral NSAIDs (35.0% vs. 25.5%; p < 0.05) and topical NSAIDs (31.1% vs. 15.9%; p < 0.0001). By contrast, RHs more frequently prescribed symptomatic slow-acting drugs for OA (SYSADOAs) (45.0% vs. 39.1%; p < 0.01) and gave more intra-articular injections of corticosteroids or hyaluronan (Table 5). GPs more frequently recommended physical therapy (p < 0.0001) and weight loss for obese patients (p < 0.0001) (Table 4).

**Table 4:** Frequency of non pharmacological and oral pharmacological treatments prescribed during the consultation for knee OA for general practitioners (GPs) and rheumatologists (RHs)

	All patients n = 1,821	GPs n = 1,570	RHs n = 251	P value
**Pharmacological treatments**				
Acetaminophen	43.4	44.0	39.8	ns
Weak opioid analgesics	30.5	30.9	28.3	ns
Strong opioid analgesics	1.9	2.0	1.2	ns
Low-dose NSAIDs	13.3	15.1	5.2	< 0.0001
Oral NSAIDs	33.7	35.0	25.5	< 0.05
Coxibs	8.5	8.9	6.4	ns
Topical NSAIDs	29.0	31.1	15.9	< 0.0001
Proton pump inhibitor	25.9	26.9	19.5	< 0.05
Symptomatic slow-acting drugs	39.9	39.1	45.0	< 0.01
Homeopathy	1.8	2.1	0.4	ns
Phytotherapy	2.9	2.7	4.4	ns
**Non pharmacological treatments**				
Weight loss (if obese)	63.4	65.4	51.4	< 0.0001
Information and education	54.6	52.3	68.9	< 0.0001
Exercise	35.7	33.9	47.4	< 0.0001
Physical therapy	21.1	33.8	21.9	< 0.001

Values are percentages. NSAIDs = nonsteroidal anti-inflammatory drugs. ns = not significant

**Table 5:** Frequency of local treatments performed during the consultation for knee OA by general practitioners (GPs) and rheumatologists (RHs)

	All patients n = 1,821	GPs n = 1,570	RHs n = 251	P value
Arthrocentesis alone	5.8	3.9	17.9	< 0.0001
Corticosteroid injection	10.8	7.6	31.5	< 0.0001
Hyaluronan injection	8.5	2.5	46.2	< 0.0001

Values are percentages.

### Correlates with prescription of low-dose NSAIDs

Prescription of low-dose NSAIDs did not depend on the presence of cardiovascular risk factors, in contrast to prescription of classical NSAIDs (p < 0.001) and coxibs (p < 0.05) (Table 6). On logistic regression analysis, prescription of low-dose NSAIDs was significantly associated with prescription by GPs (odds ratio [OR] = 2.2 [95% confidence interval [CI] 1.1-4.5]), prescription of topical NSAIDs (OR = 2.1 [95% CI 1.5-2.9]), no prescription of NSAIDs or coxibs (OR = 36.2 [95% CI 15.8-83.2] and OR = 16.0 [95% CI 4.8-52.7], respectively), and no intra-articular injections (OR = 2.2 [95% CI 1.07-4.5]).

**Table 6:** Frequency of low-dose NSAIDS, NSAIDS or coxib prescriptions according to the presence or absence of cardiovascular risk in patients with knee OA

	Absence of CV risk	Presence of CV risk	P value
Low-dose NSAIDs (n = 250)	13.2	15.2	ns
NSAIDs (n = 613)	36.7	25.8	< 0.0001
Coxibs (n = 155)	9.5	6.0	< 0.05

Values are percentages. CV = cardiovascular; NSAIDs = non steroidal anti-inflammatory drugs. ns = not significant.

## Discussion

This national observational prospective study was designed to compare the prescription patterns of GPs and RHs for their patients with knee OA, with a special emphasis on the prescription of low-dose NSAIDs.

The results of our study are consistent with published data showing that physicians in private practice in general follow the international recommendations [3,5,12] for the pharmacological treatment of OA [6,13-15]. GPs and RHs preferentially prescribed acetaminophen, and conventional NSAIDs were more frequently prescribed than were coxibs and low-dose NSAIDs. By contrast, topical NSAIDs, which should be considered ahead of oral NSAIDs according to the National Institute for Health and Clinical Excellence guidelines [5], were less frequently prescribed than were standard oral NSAIDs [6,14].

Of interest, our study identified variability by medical specialty in some aspects of the pharmacological treatment of knee OA. Indeed, GPs more frequently prescribed NSAIDs (conventional, low-dose and topical), whereas RHs more often prescribed SYSADOAs and more frequently gave intra-articular injections of steroids and hyaluronic acid. This variability is similar to what was found in the AMICA survey, which suggested that the pharmacological management of knee OA differs between GPs and RHs [6,16]. Another explanation could be the different clinical profile of patients seen by GPs or RHs. Indeed, in our study, as compared with RH patients, GP patients had longer duration of disease, more frequently had another joint affected by OA and the disease was more painful, as was previously noted in other studies [6,17]. Although in France patients can directly consult a specialist, these differences might be due to patients' more limited access to RHs, which leads patients with painful disease to consult GPs more often. Moreover, as shown in a previous British survey [17], a mixture of physical, social and psychological factors might predict visits to GPs, that may explain the different clinical profile of patients with knee OA who consulted GPs.

Our GPs and RHs did not differ in the prescription of analgesics. Acetaminophen was prescribed for only 43% of patients with chronic knee OA, which is far less that the 95.8% recorded by Denoeud et al., who assessed the pharmacological modalities prescribed by GPs as first-line treatment for knee OA [13]. This relatively low use of acetaminophen for chronic knee OA might be due to the modest efficacy of long-term intake of this drug to alleviate pain [18].

GPs and RHs did not often recommend nonpharmacological modalities such as information, physical therapy, exercise or weight loss (for obese people). For example, weight reduction, which has been showed to be effective in alleviating pain in patients with knee OA [19], was recommended by only about half of the RHs and 65% of the GPs. These figures, which are close to those obtained from a recent survey of GPs [14], highlight the need to improve the dissemination of recommendations for nonpharmacological treatment of OA [20].

Another interesting finding is the high proportion of patients with knee OA who had hypertension (57.3%) or another cardiovascular risk factor (27.5%). These results are in accordance with results of a large survey showing that 40% of patients with OA have hypertension as compared with 25% in the general population [21]. This point is of importance given the known increased cardiovascular risk associated with the use of traditional NSAIDs and coxibs, in particular for patients with established cardiovascular conditions. Of note, hypertension and diabetes mellitus were more frequent in GP patients, who were more often prescribed NSAIDs to alleviate OA pain. Cyclooxygenase inhibition causes adverse effects, in particular blood pressure elevation, mainly by impairing the systemic and renal vasodilatatory benefits of prostacyclin [22]. GPs and RHs seem to be aware of this key notion, and it affects their prescription practice because we found that NSAIDs and coxibs were prescribed less for patients with a known cardiovascular risk.

A recent large-scale study has also demonstrated a dose-dependant relation between the use of conventional NSAIDs or coxibs and risk of death and myocardial infarction in healthy individuals [9]. By contrast, use of low-dose ibuprofen was associated with decreased risk of cardiovascular events, which could be due to an antithrombotic effect comparable to that of aspirin [9].

In our survey, as compared with the prescription of classical NSAIDs and coxibs, that of low-dose NSAIDs did not depend on the perceived presence of cardiovascular risk factors and was associated with prescription by GPs, prescription of topical NSAIDs and no prescription of NSAIDs or coxibs. Thus, these practitioners prescribe low-dose NSAIDs as an alternative to conventional NSAIDs or coxibs for patients with knee OA, regardless of their cardiovascular profile.

Our study contains some limitations. Although the response rate was high (67%), participants may not have been representative of all GPs and RHs. Moreover, the proportion of GPs was much higher than that of RHs, for demographic reasons.

## Conclusions

Our study found that GPs and RHs see a different clinical profile of patients with knee OA. GP patients more often exhibit severe conditions and more often have vascular comorbidities. GPs and RHs show some variability in their management of knee OA in that GPs more frequently prescribe NSAIDs. Finally, low-dose NSAIDs, a medication prescribed more often by GPs than RHs, is used as an alternative to conventional NSAIDs or coxibs, independent of the perceived presence of cardiovascular risk factors.

## Competing interests

All authors have received fees (less than 1000 euros) from Wyeth-Santé familiale (France) as members of the scientific committee of this study named "Apaise".

## Authors' contributions

PR has written the manuscript. All authors (PR, PH, PB, PC, VB, MM) have made substantial contributions to conception and design, analysis and interpretation of data. PC and MM have performed the statistical analysis. MM has helped to draft the paper. All authors read and approved the final manuscript.

## Funding

This study was performed with the support of an independent grant provided by Wyeth-Santé Familiale France, which was not involved in the statistical analysis.

## Pre-publication history

The pre-publication history for this paper can be accessed here:

http://www.biomedcentral.com/1471-2474/12/72/prepub
